# Choledocholithiasis in Pregnancy: A Case Report

**DOI:** 10.7759/cureus.22610

**Published:** 2022-02-25

**Authors:** Fidel S Rampersad, Adrian Chan, Shirvanie Persaud, Paramanand Maharaj, Ravi Maharaj

**Affiliations:** 1 Department of Radiology, The University of the West Indies, St. Augustine, TTO; 2 Department of Surgery, Eric Williams Medical Sciences Complex (EWMSC), San Juan, TTO; 3 Department of Surgical Sciences, The University of the West Indies, St. Augustine, TTO

**Keywords:** symptomatic choledocholithiaisis, endoscopic retrograde cholangiopancreatography (ercp), magnetic resonance cholangiopancreatography (mrcp), gallstones in pregnancy, choledocholithiasis

## Abstract

Cholelithiasis during pregnancy and the postpartum period has an incidence of 12%, with pregnancy being an important risk factor for gallstones. Patients with choledocholithiasis can experience complications, such as obstructive jaundice, cholangitis, and pancreatitis, which may be detrimental to both mother and fetus.

A case of cholelithiasis in a second-trimester pregnancy was complicated by choledocholithiasis and obstructive jaundice. Ultrasonography (US), magnetic resonance cholangiopancreatography (MRCP), along with serial blood tests, confirmed the diagnosis. Treatment was safely achieved using endoscopic retrograde cholangiopancreatography (ERCP).

In pregnancy, complicated cholelithiasis is investigated using blood tests, ultrasonography, and cholangiography. Evidence supports the use of intraoperative or endoscopic cholangiography for the management of such complicated gallstone disease in pregnancy.

## Introduction

Choledocholithiasis in pregnancy is uncommon and seen in approximately one in 1200 deliveries [1]. One study showed new onset of gallbladder sludge/stone in 7.1%, 7.9%, and 10.2% of patients by the second trimester, third trimester, or postpartum period, respectively [2]. However, only 1.2% of patients with biliary sludge or stone developed symptoms attributable to gallstone disease [2]. Pregnancy is regarded as an important risk factor for gallstone disease, with data suggesting that hyperestrogenemia contributes to the saturation of bile and increasing gallbladder volume resulting in increased biliary sludge and gallstones [3-4].

Choledocholithiasis refers to the presence of gallstones in the common bile duct (CBD). Choledocholithiasis can result in complications such as obstructive jaundice, cholangitis, or pancreatitis, which can be harmful to both mother and fetus [3]. Patients with symptomatic cholelithiasis routinely have abnormal liver function tests. Radiological imaging in pregnant patients is similar to that in non-pregnant patients, with ultrasound (US) and magnetic resonance cholangiopancreatography (MRCP) being the main forms of imaging [4]. MR imaging, apart from evaluating the biliary system, can assess for evidence of gallstone pancreatitis, edema, and peripancreatic inflammatory changes. Complicated choledocholithiasis is managed definitively, with endoscopic sphincterotomy and stone extraction, along with index or scheduled cholecystectomy.

We report a case of symptomatic choledocholithiasis in a pregnant patient who underwent imaging with US and MRCP and then had a therapeutic endoscopic retrograde cholangiopancreatography (ERCP).

## Case presentation

A 22-year-old female presented to the accident and emergency department with a history of abdominal pain in the right upper quadrant (RUQ), worsening over the preceding 48 hours. She was in her seventeenth week of gestation, and this was her fourth pregnancy; with the prior three being uncomplicated. Her period of gestation was calculated using her last menstrual period, and she had not yet received any antenatal care. The patient reported intermittent and colicky right upper quadrant abdominal pain since the beginning of the current pregnancy, usually following fatty meals and resolving spontaneously. However, over the preceding two days, her pain was constant, more severe, and of longer duration. She had associated episodes of vomiting food contents. She denied any fever, chills, diaphoresis, and any changes in her stool or urine color.

On initial assessment, she was in distress from the pain. Her mucus membranes were pink, anicteric, and dry. Her vital signs remained normal and she was afebrile, with a normal respiratory rate. Abdominal examination revealed tenderness in the RUQ. She was positive for Murphy’s sign on examination but had no signs of peritonitis. A fundal height was palpated mid-way between the pubic symphysis and umbilicus.

**Figure 1:**
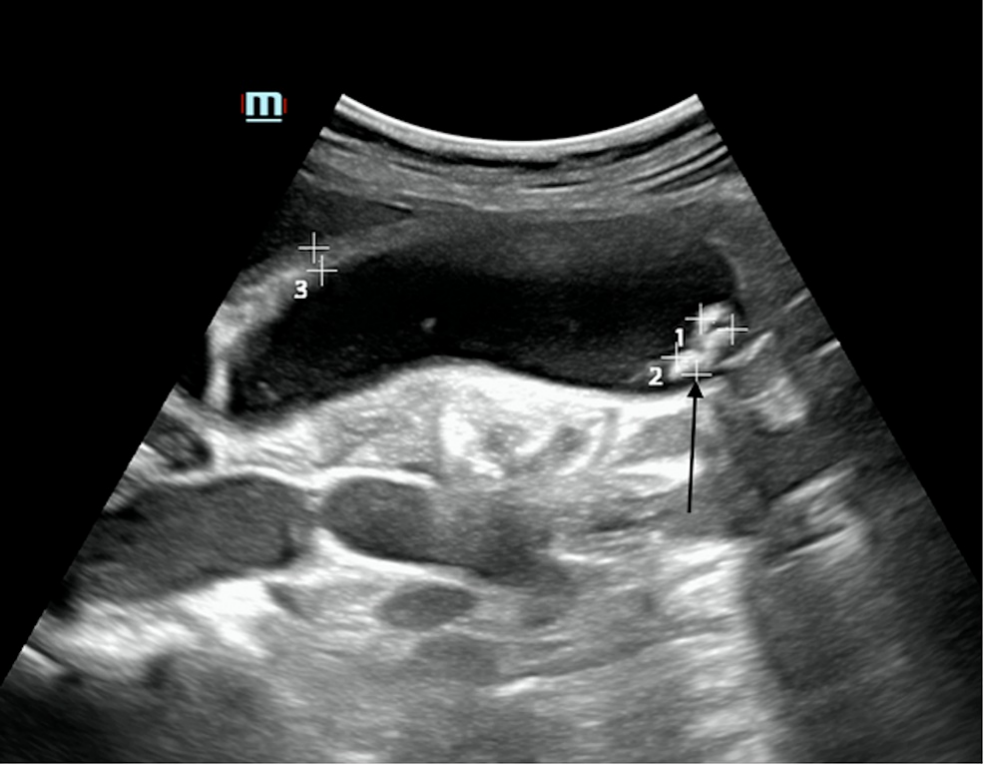
Long-axis ultrasound images of the gallbladder with multiple, well-defined hyperechoic foci and posterior acoustic shadowing in keeping with cholelithiasis (arrow) There were no sonographic features of cholecystitis.

**Figure 2:**
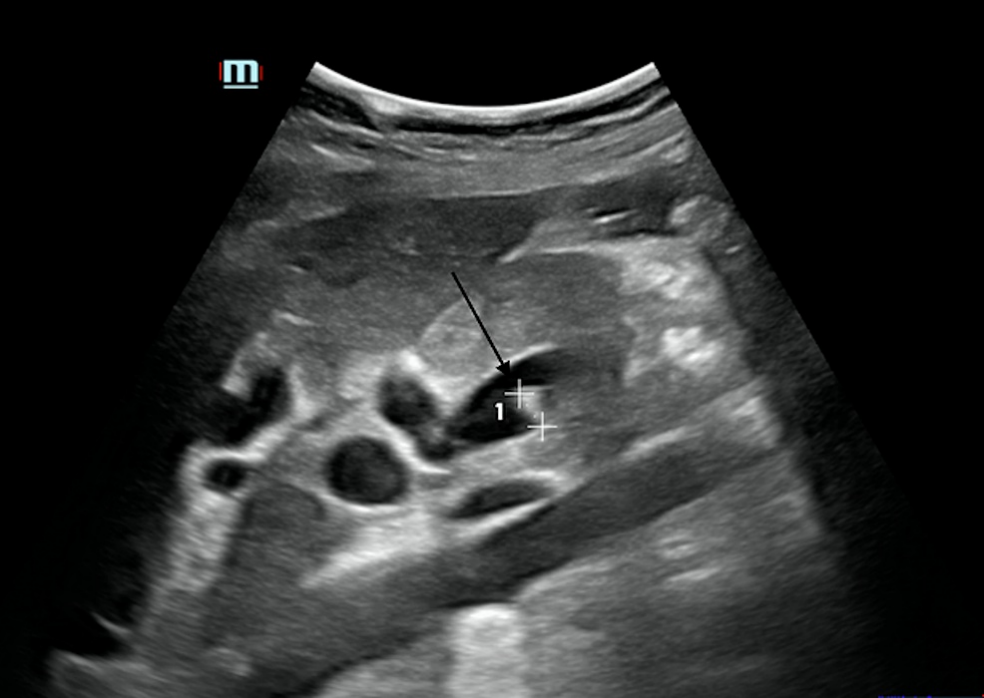
Transverse gray-scaled ultrasound of the common bile duct with evidence of a well-defined hyperechoic focus, which represents a common bile duct calculus (arrow)

The urinalysis had no abnormality. Her complete blood count had a normal leukocyte count. However, there was microcytic anemia. Her renal and liver function blood tests remained normal, including a normal partial thromboplastin time and prothrombin time. However, amylase was 673 u/L and lipase was 4163 u/L, establishing the diagnosis of gallstone pancreatitis. Fluid resuscitation was initiated using crystalloid solutions. Insertion of a Foley catheter was deferred. An abdominal US was initially performed. This revealed cholelithiasis, with no evidence of cholecystitis (Figure 1), and showed a common bile duct calculus (Figure 2).

**Figure 3:**
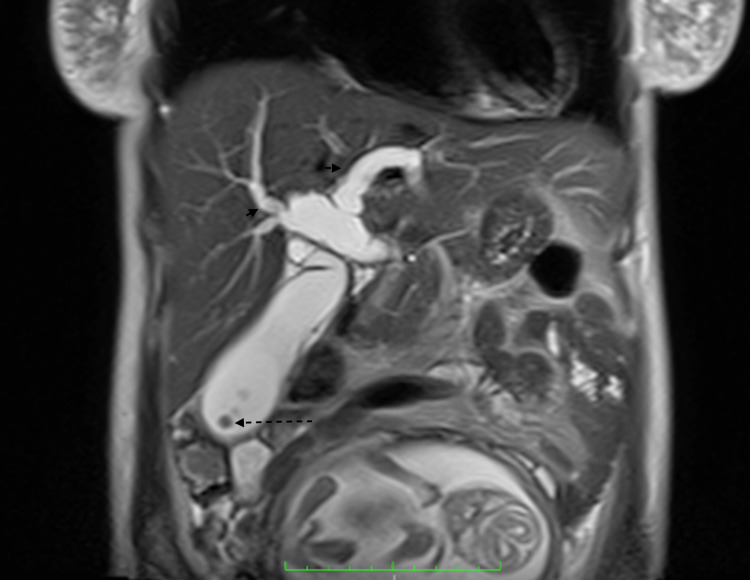
Coronal heavily T2-weighted half Fourier single-shot turbo spin-echo sequence (HASTE) demonstrates multiple well-defined low T2 signal foci (dashed arrow) representing gallstones within the distended gallbladder with intra and extra-hepatic biliary duct dilation (arrows)

**Figure 4:**
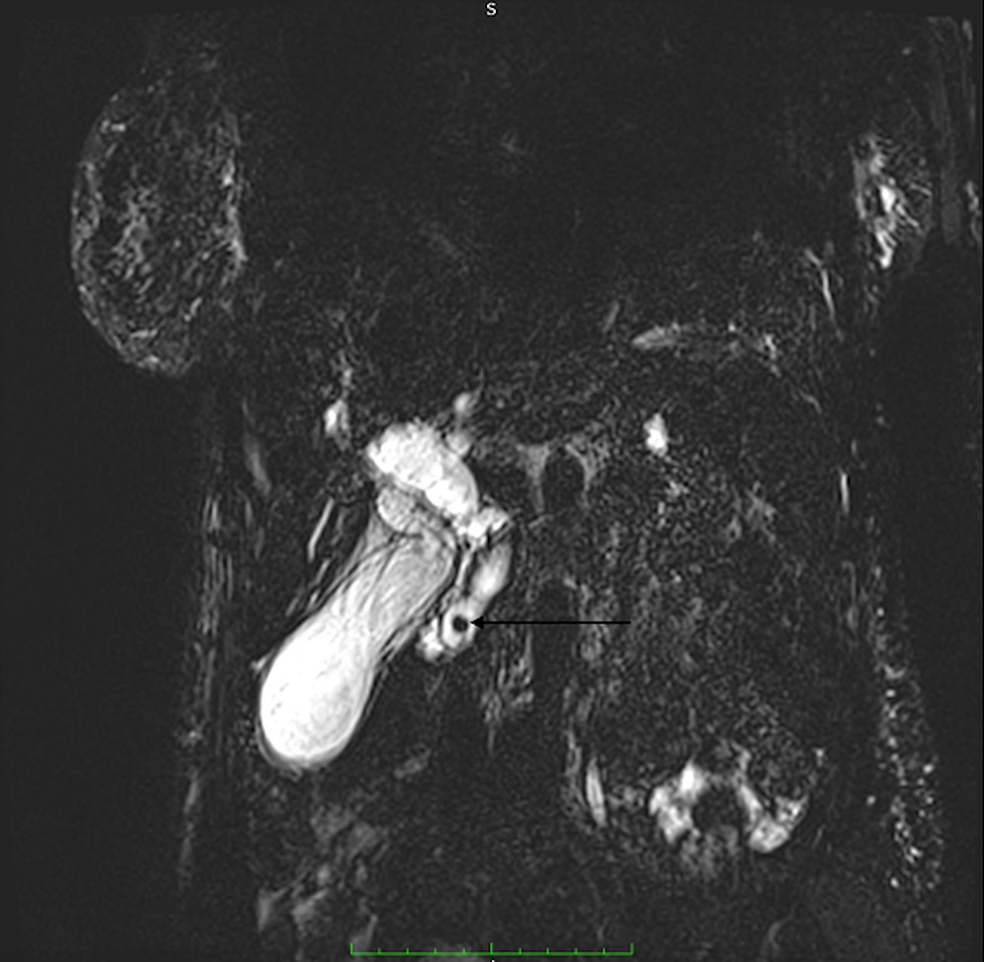
Thick-slab coronal heavily T2-weighted fast-spin echo sequence with maximum intensity projection (MIP) demonstrating a well-defined, rounded, low T2 signal focus, choledocholith (arrow) in the distal CBD with proximal intra and extrahepatic biliary duct dilatation CBD: common bile duct

With the diagnosis of choledocholithiasis and gallstone pancreatitis, this patient was kept nil per os (NPO) and closely monitored for the development of cholangitis as analgesia and fluid hydration continued. The pelvic US confirmed fetal viability. MRCP showed cholelithiasis, choledocholithiasis, and biliary duct dilatation (Figures 3-4).

The patient and her partner were counseled on the high risk of miscarriage associated with gallstone pancreatitis and choledocholithiasis. They were also counseled on the risks and benefits of ERCP, including the radiation and anesthetic risks. Consent was obtained, with the patient opting for ERCP and if it failed, for surgical CBD exploration.

**Figure 5:**
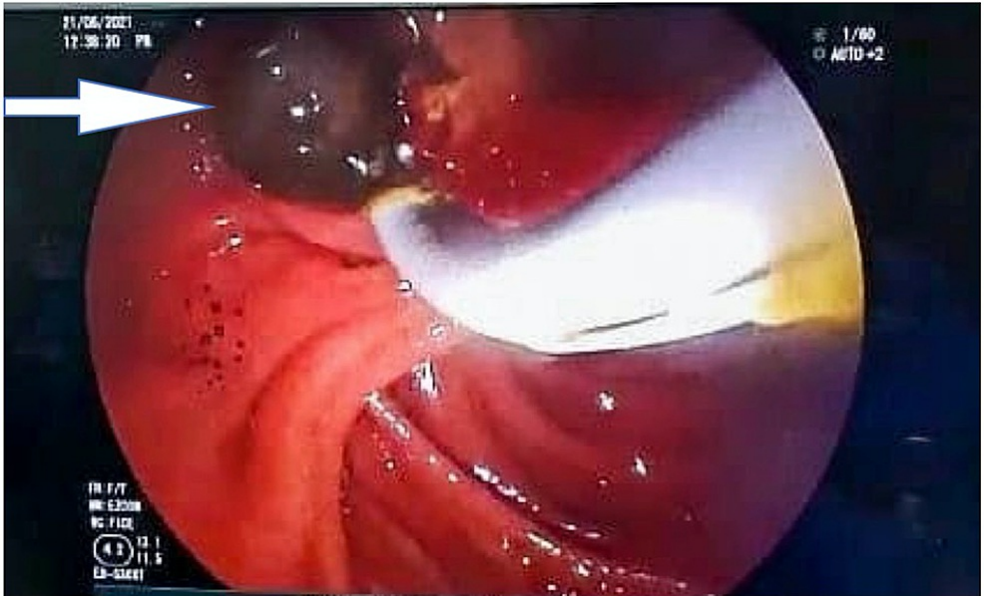
Stone (arrow) extraction after ERCP and sphincterotomy ERCP: endoscopic retrograde cholangiopancreatography

**Figure 6:**
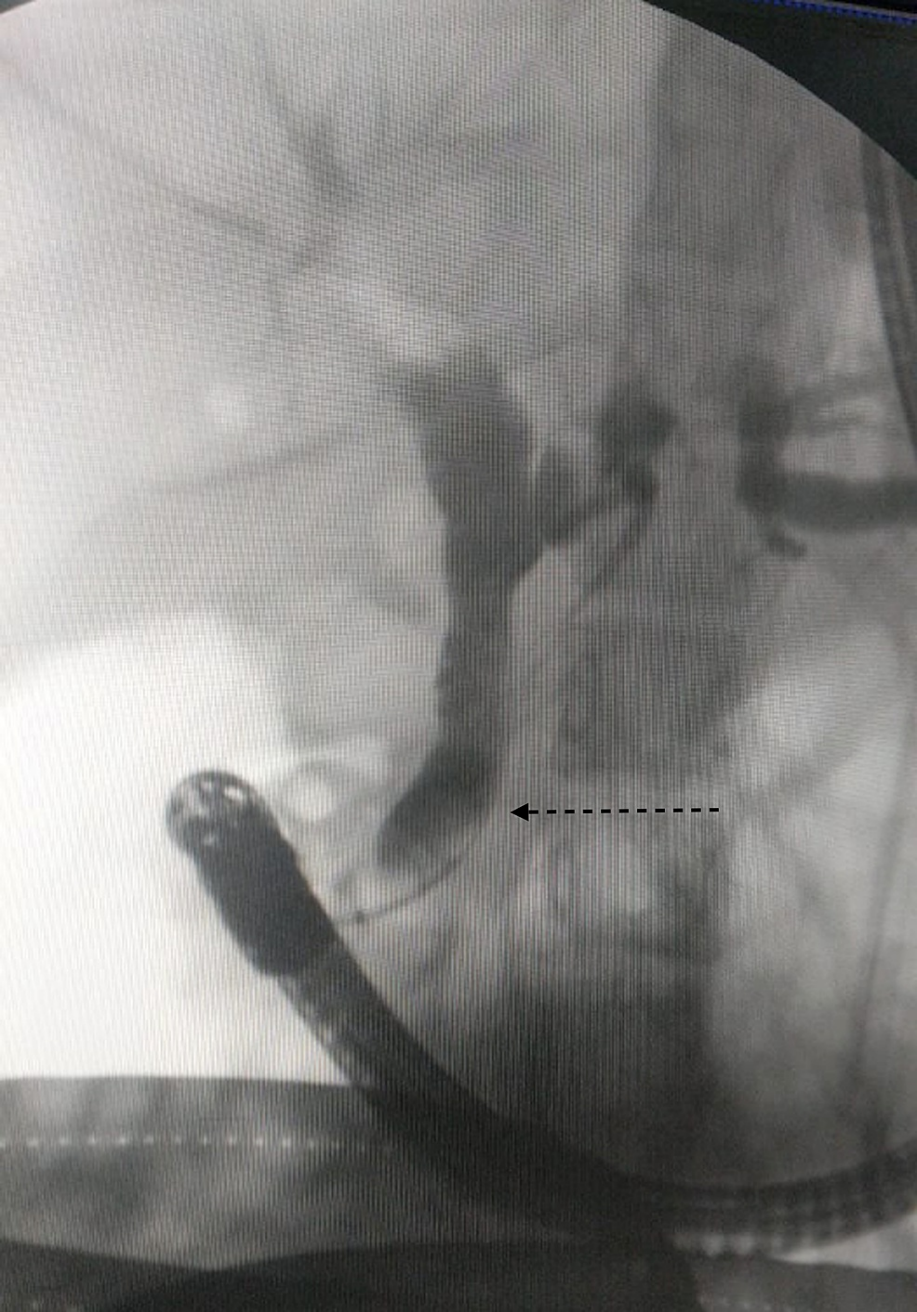
Post-procedure cholangiogram demonstrated no filling defect within the dilated CBD (dashed arrow) CBD: common bile duct

Conservative management consisted of analgesia and hydration, which was continued for the first three days of admission. The patient’s pain persisted, but she did not develop clinical features of cholangitis and her daily bilirubin checks remained within normal limits. When hyperbilirubinemia was demonstrated on Day 4 with a total bilirubin of 1.7 mg/dL (normal 0.2 to 1.3mg/dL), indirect bilirubin of 0.7mg/dL, and direct bilirubin of 0.3mg/dL; the patient was taken for ERCP. After general anesthesia, she was positioned prone, with pillows to support her hips and chest, and her lower abdomen was wrapped in the lead skirt. A side-viewing scope was passed orally and the duodenal papilla was visualized. In an effort to limit radiation exposure, the smallest possible field of the radiograph beam was utilized. The fluoroscopic screening was kept to a minimum. A 1.2 mm sphincterotomy was performed, with the passage of sludge and debris from the ampulla. A single common bile duct stone was subsequently removed (Figure 5). Post-extraction cholangiogram confirmed clearance of the biliary ducts (Figure 6).

The patient reported a significant decrease in pain after awakening from the ERCP, with the resolution of abdominal pain and the return of serum bilirubin to normal. On the next day, a normal diet was initiated. The patient was allowed home with the plan to continue antenatal care with the obstetric team that reviewed her during admission. She continued with iron supplements and prenatal vitamins. She was counseled on the risk of recurrence of complications of her cholelithiasis and possible symptoms. She will be reviewed in the surgical outpatient clinic and was scheduled for an elective laparoscopic cholecystectomy after completion of the pregnancy.

## Discussion

Choledocholithiasis in pregnancy is uncommon and seen in approximately one in 1200 deliveries [1]. In one of the largest prospective studies, with more than 3200 pregnant patients who did not have gallstones on initial US examination, new sludge/stone was noted in 7.1%, 7.9%, and 10.2% of patients by the second trimester, third trimester, or four to six-week postpartum US, respectively [2]. Pregnancy and its resultant physiologic changes cause the gallbladder volume to double, the emptying rate to slow, and motility impairment resulting in saturation of cholesterol which contributes to the ideal environment for gallstone formation [3]. One prospective study showed that only 1.2% of patients with biliary sludge or stones developed symptoms due to their disease [2]. Choledocholithiasis is defined as the presence of gallstones in the common bile duct. Its true incidence is unknown because it is most commonly asymptomatic. When symptomatic, choledocholithiasis may present as right upper quadrant pain that is more prolonged than typical episodes of biliary colic; as symptoms of obstructive jaundice such as dark urine, scleral icterus, and acholic stools; or as ascending cholangitis with Charcot’s triad of fever, right upper quadrant pain, and jaundice [3]. Progression to hypotension and mental status changes, i.e., Reynold’s pentad indicates shock from a biliary source [5]. Knowledge of the presentation of cholelithiasis and choledocholithiasis is important, as this can modulate its impact on maternal and fetal mortalities. In this case, the patient presented with right upper quadrant pain and developed obstructive jaundice.

The aim of imaging in the pregnant patient is to differentiate choledocholithiasis from intrahepatic cholestasis of pregnancy, as both clinical and biochemical presentations are similar. Ultrasonography and MRCP are the two main modalities of imaging of suspected choledocholithiasis in pregnancy [4-5]. Ultrasonography is regarded as the initial modality of choice to evaluate the hepatobiliary system in pregnancy, as it is safe, low cost, reliable, and readily available. However, limitations include shadowing due to overlying bowel gas, poor beam penetration due to body habitus, and the fact that it is operator-dependent [4]. In this case, the US was proven useful in the initial evaluation, with the identification of gallstones within the gallbladder and CBD. Ultrasonography and MRI are excellent to use in pregnancy, as they can provide multiplanar imaging in the absence of ionizing radiation. In contrast, MRI offers increased sensitivity in detecting diffuse liver disease, improved spatial resolution, and an increase in soft-tissue contrast, allowing for its large fields of view (FOVs). Relevant to this case, MRCP has a specificity of 93% and sensitivity of 88% with detailed evaluation of the biliary tree; it allows further evaluation of the pancreas with detection of edema, obstruction of the pancreatic duct in gallstone pancreatitis, and surrounding inflammatory changes [6-8].

Supportive care is an important aspect of the management of choledocholithiasis [9]. Analgesia, antibiotics, and intravenous fluids are prescribed. Intraoperative and postoperative pneumatic compression devices and early postoperative ambulation are done as prophylaxis against deep venous thrombosis in the pregnant patient.

Choledocholithiasis in pregnancy can be managed safely with ERCP and sphincterotomy followed by laparoscopic cholecystectomy [10-12]. Laparoscopic or open CBD exploration and index cholecystectomy are also acceptable. Contemporary literature supports that laparoscopic surgery performed during any trimester is safe for the mother and fetus however laparoscopic surgery was not readily available in our facility due to lack of the equipment needed to safely perform laparoscopic surgery in the current coronavirus disease 2019 (COVID-19) pandemic [13]. The endoscopic methods for bile duct clearance include sphincterotomy, balloon dilation of the ampulla, and basket or balloon extraction. Sphincterotomy involves severing the deep muscle layers of the sphincter of Oddi with electrocautery. It should be performed by high-volume practitioners to improve success rates and to minimize procedure duration and radiation exposure [14]. The usual complications of ERCP, including post-sphincterotomy bleeding, pancreatitis, and perforation, can have greater consequences in a pregnant woman. A meta-analysis of 27 studies assessed the safety of ERCP in pregnancy included 1,307 patients. The overall adverse event rate was 15.9 % [15].

Cumulative radiation dosage should be limited to 50-100 milliGray (mGy) during pregnancy to decrease the risk of teratogenesis and childhood leukemia [16-17]. Radiation exposure during intra-operative cholangiography is estimated to be 20-50 mGy. The radiation exposure during ERCP averages 20-120 mGy [18]. Efforts should be made to shield the fetus from radiation exposure without compromising the field of view. The lead apron shield must be placed underneath the patient and not simply draped over the abdomen since the radiation source is underneath the patient when using the standard fluoroscopy C-arm. Endoscopic ultrasound (EUS) provides an alternative (non-ionizing) means of imaging the CBD. Transabdominal US-guided non-radiation ERCP procedures for the removal of CBD stones during pregnancy have been demonstrated [19]. More studies are needed to assess its safety and efficacy.

Non-radiation ERCP is a sophisticated procedure that requires a high level of expertise [14]. Successful bile-duct cannulation with sphincterotomy and clearance of biliary stones or sludge was performed without fluoroscopy in a series of 21 pregnant women. One case of mild post-ERCP pancreatitis was the only reported adverse event. Choledochoscopy confirmed ductal clearance in five cases [20]. Noteworthy, there was no significant difference between the subgroups of radiation-ERCP and non-radiation ERCP in terms of fetal outcomes (5.2 % versus 6.2 %). Unexpectedly, maternal non-pregnancy-related complications occurred half as often in the non-radiation group than conventional radiation ERCP (7.6 % versus 14.9 %). This may have been due to extensive pre-procedure workup, along with the need for expert endoscopists to perform non-radiation ERCP [15].

Another concern is with anesthesia during endoscopy. The medications used for sedation are poorly studied in pregnancy but the current recommendation is to use the lowest effective dose of category B drugs [14]. Monitoring is done with continuous electrocardiography, pulse oximetry, and intermittent sphygmomanometry [21]. In patient positioning, care must be taken to avoid the physiological changes of uterine compression of the inferior vena cava. The index patient was positioned prone. However, acceptable positioning also includes left pelvic tilt or left lateral position [14].

If ERCP is not possible, percutaneous transhepatic biliary drainage (PTBD) serves as an option for biliary decompression. Additionally, CBD exploration via choledochotomy with stone removal with or without T-tube placement allows biliary decompression.

If cholelithiasis is present, an index cholecystectomy can be performed in a stable patient. Scheduling cholecystectomy until after delivery is associated with high rates of recurrent symptoms, emergency department visits, and recurrent hospitalizations [22]. As seen in this case, ERCP and sphincterotomy may be sufficient to prevent recurrence during pregnancy [23]. Complicated gallstone disease results in preterm labor and fetal loss. Optimal management is, therefore, necessary for fetal and maternal well-being.

## Conclusions

Imaging of suspected choledocholithiasis in pregnancy can be safely performed with ultrasound and MRCP, the latter being better-suited to evaluate the CBD and provide information regarding the size, number, and position of gallstones within the CBD. Choledocholithiasis in pregnancy can be safely managed with ERCP and sphincterotomy. This can be followed by an index laparoscopic cholecystectomy; otherwise, the risk of recurrent complications remains high. Cholangiography (intraoperative or endoscopic) can be used selectively during pregnancy because of its adequately low radiation exposure to the mother and fetus, especially when the lower abdomen is shielded.
